# Factors associated with low birth weight in Nepal using multiple imputation

**DOI:** 10.1186/s12884-017-1252-5

**Published:** 2017-02-20

**Authors:** Usha Singh, Attachai Ueranantasun, Metta Kuning

**Affiliations:** 1 Nepal Institute of Health Sciences, Gokarneswor Municipality-12, Jorpati, Kathmandu, Nepal; 2 Department of Mathematics and Computer Science, Faculty of Science and Technology, Prince of Songkla University, Pattani Campus, Pattani, 94000 Thailand

**Keywords:** Multiple imputation, Low birth weight, Survey package and Transform-then impute

## Abstract

**Background:**

Survey data from low income countries on birth weight usually pose a persistent problem. The studies conducted on birth weight have acknowledged missing data on birth weight, but they are not included in the analysis. Furthermore, other missing data presented on determinants of birth weight are not addressed. Thus, this study tries to identify determinants that are associated with low birth weight (LBW) using multiple imputation to handle missing data on birth weight and its determinants.

**Methods:**

The child dataset from Nepal Demographic and Health Survey (NDHS), 2011 was utilized in this study. A total of 5,240 children were born between 2006 and 2011, out of which 87% had at least one measured variable missing and 21% had no recorded birth weight. All the analyses were carried out in R version 3.1.3. Transform-then impute method was applied to check for interaction between explanatory variables and imputed missing data. Survey package was applied to each imputed dataset to account for survey design and sampling method. Survey logistic regression was applied to identify the determinants associated with LBW.

**Results:**

The prevalence of LBW was 15.4% after imputation. Women with the highest autonomy on their own health compared to those with health decisions involving husband or others (adjusted odds ratio (OR) 1.87, 95% confidence interval (95% CI) = 1.31, 2.67), and husband and women together (adjusted OR 1.57, 95% CI = 1.05, 2.35) were less likely to give birth to LBW infants. Mothers using highly polluting cooking fuels (adjusted OR 1.49, 95% CI = 1.03, 2.22) were more likely to give birth to LBW infants than mothers using non-polluting cooking fuels.

**Conclusion:**

The findings of this study suggested that obtaining the prevalence of LBW from only the sample of measured birth weight and ignoring missing data results in underestimation.

## Background

Missing data occur almost in all types of studies and cause inefficient and biased estimates of parameters if they are handled improperly. In a survey, missing data occur, when a selected respondent refuses to participate (unit nonresponse) or respondent does not provide answer to entire survey questions (item nonresponse) [1, 2]. For unit nonresponse, the weighting adjustment technique is applied, in which weight of respondents are increased to represent non-respondents [3], whereas for item nonresponse, imputation methods are employed [1, 4].

There are three types of mechanisms under which missing data occur: missing completely at random (MCAR), missing at random (MAR) and missing not at random (MNAR) [1, 5]. When missing data are MCAR, the probability of missingness does not depend on the missing and other observed data. An example is when survey papers are lost accidentally. If missing data are MAR, the probability of missingness depends only on observed data, but not on the missing data themselves. For example, people from different demographic backgrounds may decline to answer based on beliefs or traditions. When missing data are MNAR, the probability of missingness depends on both observed and missing data. For example, people with high incomes are less likely to report their incomes than those of people with average or low income. Data under MCAR mechanism can be tested statistically by little’s test [6]. However, there is no clear technique to diagnose and distinguish between MAR and MNAR. Thus, MAR and MNAR can only be reasoned or hypothesized [1, 4].

There are several studies about methods used for handling missing data in each type of missing mechanisms [7]. The most common method is case deletion in which subjects with missing values are deleted. The results from this method are inefficient, but unbiased, when the missing data hold MCAR assumption. However, when data are not MCAR, the results from this method are inefficient and biased [4, 5]. Methods like mean substitution, last observation carried forward, hot deck imputation, cold deck imputation and regression imputation come under single imputation in which missing values are replaced by synthetic values [2, 8]. The first two methods of single imputation assume missing data are MCAR, while the remaining methods assume missing data are MAR [7]. The results obtained from mean substitution and hot deck imputation are biased under three missing mechanisms. However, the results obtained from conditional mean imputation are unbiased under MCAR and MAR, but may be biased under MNAR [4]. Furthermore, in single imputation, values are imputed for one time; the uncertainties created by missing values are not accounted for. As a result, there are small standard errors, p-values and narrow confidence intervals [5, 9]. In multiple imputation, unlike single imputation, missing values are imputed for more than one time and the uncertainties created by missing values are incorporated resulting in larger standard errors and wider confidence intervals [1]. In addition, multiple imputation provide unbiased result, when data hold both MAR and MNAR assumption [4].

In southern Asia and sub-Saharan Africa, more than half of women give birth at home [10]. Therefore, analyzing data on infants delivered only at hospital would be biased [11]. As a substitute to hospital based data, household data survey begin to collect information on infants born outside health facilities [12]. However, the data on birth weight from a household survey become limited since mothers are unable to provide numeric birth weight [11, 12]. Nepal Demographic and Health Survey (NDHS), 2011 reported that only 36% of weights of infants were measured at the time of birth [13]. The same survey also reported that the prevalence of low birth weight (LBW) in Nepal was 12%, which was calculated from the available birth weight of infants. Studies conducted in Nepal on LBW by using demographic and health survey (DHS) data either have considered mother’s recall for infant’s size at birth as an alternative to the birth weight [14] or analyzed the subset of measured birth weight [15] for identifying the prevalence and factors associated with LBW. Estimating prevalence of LBW and identifying determinants associated with it only from the available birth weight may be biased, when missing birth weight are not MCAR. Besides missing values on the birth weight, missing values are also presented on determinants of birth weight, but are not handled in most of previous studies and the results obtained from these studies may be misrepresented. Thus, the main objective of this study is to identify factors associated with LBW using multiple imputation to handle missing data in both outcome and determinants.

## Methods

### NDHS data

The child dataset from Nepal Demographic and Health Survey (NDHS), 2011 was analyzed in this study. NDHS is a nationally representative household survey conducted every 5 years [13]. Multistage cluster sampling was used in this survey. In the first stage, the probability proportionate to size was used to select wards from rural areas and sub-wards from urban areas. In the second stage, random sampling was done to select households [13, 16]. Details of clustering, listing and sample selection have been mentioned elsewhere [16]. The survey interviewed 12,674 women aged 15 to 49 and 4,121 men aged 15 to 59. Three main questionnaires were administered including household questionnaire, women’s questionnaire and men’s questionnaire to collect information from different levels. These questionnaires contained different units of analysis and they were eventually converted into seven datasets [13]. In this study, child dataset was used. From this dataset, a total of 5,306 children were born during the period of 2006–2011. Children from multiple births tend to have lower birth weights than singletons[17]. Thus, 66 multiple births were excluded from this study and only 5,240 singleton children were included in this study. However, out of 5,240 children, 766 (13.4%) children had completed the record and 4,474 (86.6%) children had at least one of the measured variables missing.

### Study variables

Birth weight of an infant was considered the outcome of this study. Based on World Health Organization (WHO) classification, birth weight was divided into normal birth weight, equal to or greater than 2,500 g, and LBW, lower than 2,500 g [18]. In this study, all the study variables that were included in [15] were employed. The study variables were classified under three major determinants. These are underlying factors, proximate factors and factors related to gestation and fetal growth. The underlying factors were made up of economic status (wealth index), mother’s education, women’s decision for utilization of health services, ethnicity, residence and development region. Body mass index (BMI), birth interval, antenatal care (ANC) visits, and consumption of iron tablets during pregnancy, smoking and use of polluting cooking fuel constituted the proximate factors. Gestation and fetal growth factors were mother’s age at child’s birth, parity and gender of child were employed in this study. Besides birth weight, NDHS 2011 asked a specific question to mothers about the size of their babies at the time of birth. Based upon five categories namely very large, large, normal, small and very small, mothers had to recall their babies’ size. This variable was used as an auxiliary variable for imputing birth weight in this study. In the dataset, there was no mother’s age at child’s birth variable. Mother’s age at child’s birth was calculated from mother’s current age minus date of child’s birth and was categorized as 15–19 years, 20–24 years, 25–29 years and 30 years and above. Mother’s education was categorized into no education, primary education, secondary/higher education. Here, all study variables were in categories and the categorization of the study variables were based on previous studies which used similar DHS datasets conducted in Nepal [14, 15].

### Frequency, pattern and reason for missingness of missing data

**Fig. 1:**
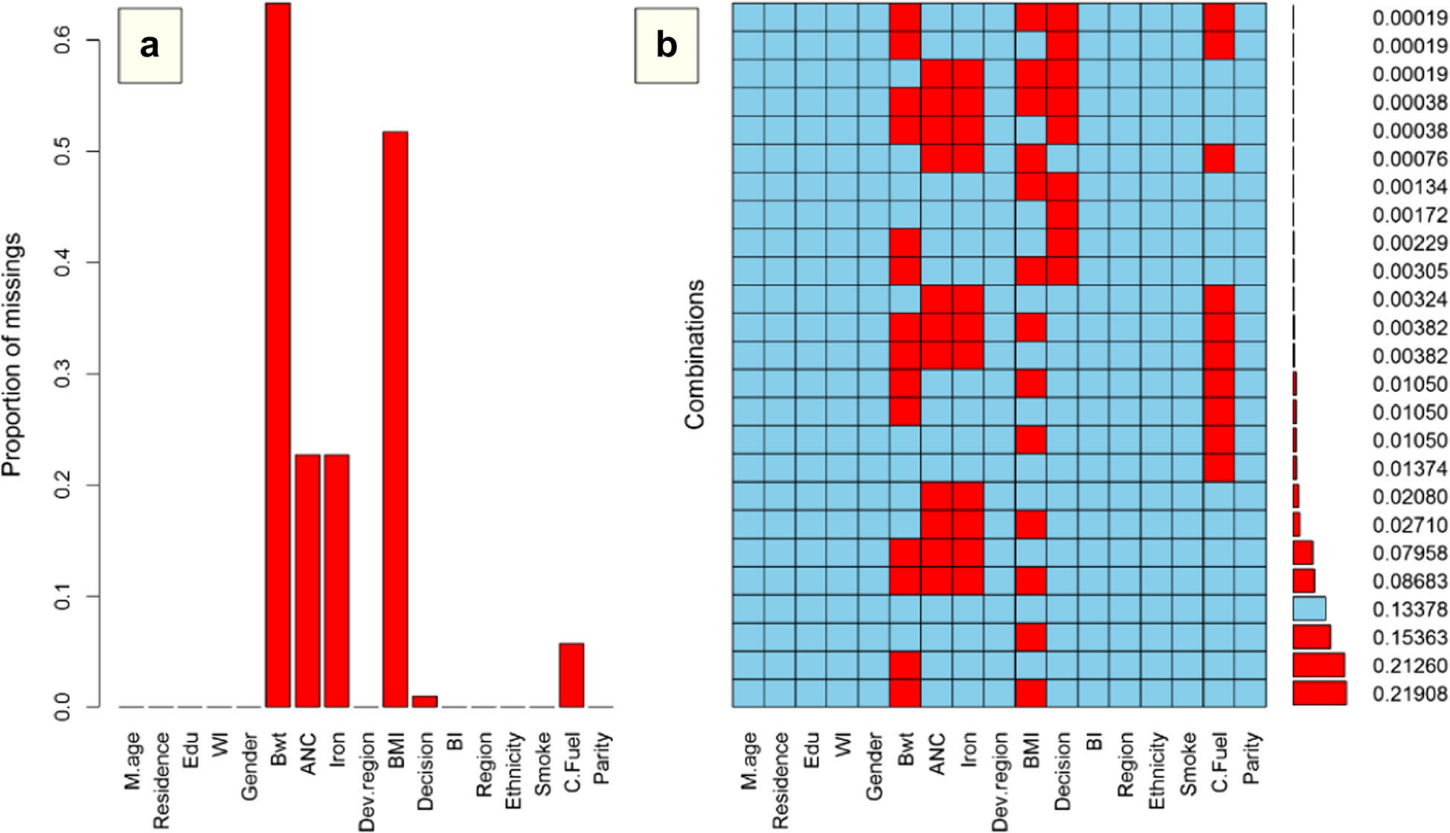
Percentage and pattern of missing data. Note: *M.age: Mother’s age at child’s birth, Edu: education, WI: wealth index, Bwt: birth weight, Iron: consumption of iron tablets during pregnancy, Decision: women’s decision for utilization of health services, BI: birth interval and C. Fuel: cooking fuel*

Before handling missing data, the frequency, pattern and reason for missing data were checked. For the graphical presentation of missing data and its pattern, VIM package in R was used. The percentages of missing values in each variable and the patterns of missing data are displayed in Fig. 1, left (a) and right (b) panel respectively. From Fig. 1a, the highest percentages of missing values were from the variables of birth weight (63.32%), and BMI (52%). The percentages of missing were nearly equal for ANC and consumption of iron tablets during pregnancy, whereas the percentages of missingness were less than 10 for cooking fuel and women’s decision for utilization health of services. There were no missing values for the variables such as mother’s age at child’s birth, gender of child, parity, mother’s education, wealth index, ethnicity, residence, ecological region, development region, birth interval and smoking. The pattern of missing data shown in Fig. 1b was arbitrary, because the missing values for the variables of any record were seen in a random fashion. From Fig. 1b, only 13.4% of children had completed the record without missing values, while 21.2% of children data contained missing values only on birth weight and 15.4% children had missing values only on BMI. Furthermore, 21.9% of children had missing values on both birth weight and BMI, and only 8.7% of infants data contained missing values on birth weight, mother’s BMI, ANC visit and consumption of iron tablets during pregnancy.

The missing mechanism was diagnosed by implementing Little’s test [6] to identify whether missing data were MCAR. The test revealed that data were not MCAR since *p*-value was found to be around 0.000. From the data, missing birth weight was due to home delivery. The reason for missing values on ANC visit was probably because mothers living in rural areas felt shy to report a number of ANC visits during the time of interview. Missing values on consumption of iron tablets during pregnancy might be due to missing values on ANC visit, because mothers who did not report their ANC visit were less likely to report any consumption of iron tablets during pregnancy. Missing values on mother’s BMI were because of refusal to measure height and weight either by a respondent or a respondent’s mother. Furthermore, missing values for cooking fuel were for those mothers who did not belong to the household (non de jure residents), but presented at the place for the time of an interview. It was evident that missing values in the variables were not missing due to themselves, but were missing due to other characteristics. Thus, in this study, missing data were under MAR assumption.

### Background of Multiple imputation

Multiple imputation yields unbiased estimates of parameters, when missing data hold MAR assumption [4], and as aforementioned in the last section, missing data in this study held MAR assumption. Therefore, multiple imputation was applied to handle missing data. In multiple imputation technique, each missing value is imputed by *m* > 1 times resulting into *m* datasets. Each dataset is analyzed by using complete data method. The estimates of parameters of *m* datasets are pooled to calculate overall estimates of parameters and confidence intervals that identify missing data uncertainty [1].

For combining estimates of parameters of *m* datasets, formulas derived by [1] is used. Suppose the regression coefficient for an imputed dataset *i* is *Q*_*i*_and*U*_*i*_ be the variance where *i* = 1, 2,…,m. Therefore, the overall regression coefficient is the average of all *Q*_*i*_ and shown in Eq. (1).

1 $$ \overline{Q}=\frac{1}{m}{\displaystyle {\sum}_{i=1}^m{Q}_i} $$

The variance within imputation is average of all*U*_*i*_ and is shown in Eq. (2).

2 $$ \overline{U}=\frac{1}{m}{\displaystyle {\sum}_{i=1}^m{U}_i} $$

The variance between imputations is displayed in Eq. (3).

3 $$ B=\frac{1}{m-1}{\displaystyle {\sum}_{i=1}^m{\left({Q}_i-\overline{Q}\right)}^2} $$

The total variance is a combination of variance within and in between imputations which is displayed in Eq. (4).

4 $$ T=\overline{U}+\left(1+\frac{1}{m}\right) B $$

The overall standard error is the square root of total variance T and is displayed in Eq. 5.

5 $$ S E=\sqrt{T} $$

In multiple imputation, methods like Joint Modeling (JM) and Multiple Imputation by Chained Equations (MICE) also called as Fully Conditional Specification (FCS) have been proposed to impute missing data [19]. In MICE approach, a series of regression models are performed in which each variable with missing data is modeled conditionally upon other variables in the dataset. This signifies that each variable has its own imputation model. For example, logistic regression model is used for binary variables and linear regression model used for continuous variables [20]. As described in [19], multiple imputation involves three main steps: imputation, analysis and pooling. Firstly, an imputation model is used to generate the missing values using possible values. In the imputation model, auxiliary variables and variables that can explain a missing mechanism are kept for a better prediction of missing values and making MAR hypothesis more possible [21, 22]. Initially, three to five imputations are suggested for obtaining outstanding results [23]; however, [24] recommended the number of imputation should be over than or equal to the percentage of missing data. Secondly, an analysis model is applied to estimate parameters for each imputed dataset. Basically, in theory, the analysis model and the imputation model need to be the same, but they can be different in practice [22]. Finally, the estimated coefficients, standard errors and confidence intervals from each model are pooled together using Rubin’s rule.

In this study, missing values were in both independent and dependent variables. As stated by [6], if the missing values are presented in both determinants (X) and outcome (Y), then cases with the missing outcome (Y) can confer a little information for the regression of interest, by improving prediction of missing determinants (X) for cases with the outcome (Y) present. Therefore, under a particular condition Multiple Imputation then Deletion (MID) performs better than standard multiple imputation in which all missing values on determinants (X) and outcome (Y) are imputed, and then deleting cases with imputed values on outcome (Y) before analysis [25]. However, standard multiple imputation performs better than MID when auxiliary variables are included in an imputation model as stated by [26]. Hence, mother’s opinion on infant’s size at birth was employed as an auxiliary variable in this study for the better result.

### Implementation and statistical analysis

The pattern of missing data in this study was arbitrary and missing variables were categorical; hence, FCS method was considered appropriate [19]. Therefore, mice package in R was used in this study, because in mice package, multiple imputation using FCS is implemented by MICE algorithm [19]. For combining each imputed dataset, mitools package by [27] was applied. In this study, multiple imputation was carried out for 65 times, because the highest percentage of missing was 63.32. As suggested by [28], mother’s opinion on infant’s birth size can be used as an alternative to the birth weight. Therefore mother’s opinion on infant’s size at birth was considered an auxiliary variable in this study. Before the imputation model, possible interaction between the variables like ANC and iron tablets consumption during pregnancy, wealth index and mother’s education, education and women’s decision for utilization of health services, ecological region and developmental region, and development region and women’s decision for utilization of health services was checked using transform-then-impute method as described by [29]. It was found that no interaction among them presented, because the *p*-value was greater than 0.05. Consequently, all the study variables along with auxiliary variable were included into the imputation model. Survey package by [30] in R was applied to each imputed dataset to account for sampling method and sample weights. Survey logistic regression model as an analysis model was applied to identify the factors associated with LBW. Under complex survey data, the parameters are estimated by pseudo likelihood method instead of maximum likelihood [31]. Therefore, the adjusted Wald test statistic was applied for selecting significant variables.

## Results

### Prevalence of LBW

The overall and crude subgroup estimations of LBW prevalence and their 95% confidence intervals (95% CI) were calculated and shown in Table 1. The overall prevalence of LBW was 15.4% (95% CI = 12–18%) after imputation. The prevalences of LBW for the determinants like residence, ethnicity, mother’s age at child’s birth, parity and gender of child were nearly equal in each subgroup. However, the prevalence of LBW was different in each subgroup for the rest of variables. For the variables such as wealth index, BMI and ANC visit, the percentages of LBW were showing a decreasing trend starting from poor, underweight and no ANC visit respectively. For ecological region, the lowest prevalence of having LBW babies was for mothers living in Terai (13.7%), while mothers from other two subgroups had nearly similar prevalences of having LBW babies. Likewise, the percentage of giving birth to LBW infants was the highest for mothers who gave birth to infants within a gap of less than 24 months from the previous birth (19.1%), while mothers for other two subgroups had almost equal prevalences of giving birth to LBW infants. For mothers who were not consuming iron tablets during pregnancy (18.9%), being smoker (21.0%) and using highly polluting cooking fuel (16.1%) showed the highest prevalences of having LBW babies compared to their respective subgroups. The percentages of giving birth to LBW infants among mothers who attended primary education (18.3%) and no education (16.1%) were close and higher than uneducated mothers (16.1%). In case of development region, the higher prevalences were evident in mothers residing in Far-western (19.2%), Eastern (18.3) and Mid-western (17.4%) than in mothers residing in other development regions.

**Table 1:** Overall and subgroup prevalences of LBW after imputation

Variables	Estimate	SE	95% CI
Overall	0.154	0.015	0.12, 0.18
Underlying factors			
Wealth index			
Poor	0.174	0.024	0.13, 0.22
Middle	0.152	0.026	0.10, 0.20
Rich	0.123	0.014	0.10, 0.15
Mother’s education			
No education	0.161	0.025	0.11, 0.21
Primary education	0.183	0.022	0.14, 0.23
Secondary/higher education	0.124	0.014	0.10, 0.15
Women’s decision for health service utilization			
Women	0.105	0.016	0.07, 0.14
Women and husband together	0.152	0.019	0.11, 0.19
Husband or others	0.181	0.020	0.14, 0.22
Ethnicity			
Relatively advantaged	0.159	0.018	0.12, 0.19
Relatively disadvantaged (Janjati)	0.142	0.020	0.10 0.18
Relatively disadvantaged (Dalit)	0.160	0.024	0.11, 0.21
Residence			
Rural	0.155	0.016	0.13, 0.19
Urban	0.140	0.014	0.11, 0.17
Ecological region			
Mountain	0.178	0.033	0.11, 0.24
Hill	0.170	0.020	0.13, 0.21
Terai	0.137	0.018	0.10, 0.17
Development region			
Eastern	0.183	0.024	0.14, 0.23
Central	0.121	0.021	0.08, 0.16
Western	0.131	0.025	0.08, 0.18
Mid-western	0.174	0.025	0.12, 0.22
Far-western	0.192	0.029	0.13, 0.25
Proximate factors			
Body mass index (BMI)			
< 18.5 (Underweight)	0.176	0.028	0.12, 0.23
18.5–23.0 (Normal)	0.160	0.018	0.12, 0.19
> 23.0 (Overweight)	0.115	0.021	0.07, 0.16
Birth interval			
No interval	0.150	0.014	0.12, 0.18
< 24 months	0.191	0.037	0.12, 0.26
≥ 24 months	0.146	0.019	0.11, 0.18
ANC visit during pregnancy			
No visit	0.207	0.048	0.11, 0.30
One-three visits	0.167	0.023	0.12, 0.21
Four or more visits	0.125	0.012	0.10, 0.15
Consumption of iron tablets during pregnancy			
No	0.189	0.036	0.12, 0.26
Yes	0.143	0.013	0.12, 0.17
Smoking			
No	0.149	0.014	0.12, 0.18
Yes	0.210	0.058	0.10, 0.32
Fuel			
Low polluting fuel	0.110	0.017	0.08, 0.14
Highly polluting fuel	0.161	0.016	0.13, 0.19
Gestation and fetal growth factors			
Mother’s age at child’s birth (Years)			
15–19	0.160	0.020	0.12, 0.20
20–24	0.149	0.016	0.12, 0.18
25–29	0.149	0.022	0.11, 0.19
≥ 30	0.162	0.034	0.09, 0.23
Parity			
One	0.140	0.015	0.11, 0.17
Two-three	0.154	0.014	0.13, 0.18
Four and above	0.164	0.034	0.10, 0.23
Gender of baby			
Male	0.146	0.017	0.11, 0.18
Female	0.161	0.017	0.13, 0.20

### Factors associated with LBW

For the univariate analysis, all study variables were analyzed by using simple survey logistic regression and results are displayed in Table 2. Women’s decision for utilization of health services and cooking fuel were found statistically significant. Mothers were more likely to give birth to LBW infants, when decision on utilization of health services relied on husband and others (OR 1.91, 95% CI = 1.34, 2.72) and mother and her husband together (OR 1.54, 95% CI = 1.03, 2.30). Mothers using highly polluting cooking fuels (OR 1.56, 95% CI = 1.07, 2.28) were more likely to give birth to LBW infants than mothers using non-polluting cooking fuels. However, the variables like wealth index, mother’s education, ethnicity, residence, ecological region, developmental region, mother’s BMI, birth interval, ANC visit, consumption of iron tablets during pregnancy, smoking, mother’s age at child’s birth, parity and gender of child remain insignificant with LBW.

**Table 2:** Unadjusted odds ratio and 95% CI of study variables

Variables	Unadjusted OR	95% CI	*p*-value
Underlying factors			
Wealth index			
Rich	1.00		0.107
Middle	1.27	0.83, 1.96	
Poor	1.50	1.03, 2.20	
Mother’s education			
Secondary/higher education	1.00		0.107
Primary education	1.58	1.09, 2.27	
No education	1.34	0.90, 2.00	
Women’s decision for health service utilization			
Women	1.00		0.002*
Women and husband together	1.54	1.03, 2.30	
Husband or others	1.91	1.34, 2.72	
Ethnicity			
Relatively advantaged	1.00		0.758
Relatively disadvantaged (Janjati)	0.88	0.61, 1.26	
Relatively disadvantaged (Dalit)	1.00	0.67, 1.49	
Residence			
Urban	1.00		0.466
Rural	1.12	0.82, 1.53	
Ecological region			
Terai	1.00		0.289
Hill	1.29	0.89, 1.87	
Mountain	1.37	0.87, 2.17	
Development region			
Central	1.00		0.072
Eastern	1.63	1.03, 2.57	
Western	1.09	0.64, 1.84	
Mid-western	1.53	0.92, 2.57	
Far-western	1.72	1.07, 2.78	
Proximate factors			
Body mass index (BMI)			
> 23.0 (Overweight)	1.00		0.138
18.5–23.0 (Normal)	1.50	0.96, 2.33	
< 18.5 (Underweight)	1.67	1.03, 2.71	
Birth interval			
No interval	1.00		0.338
< 24 months	1.32	0.82, 2.15	
≥ 24 months	0.96	0.69, 1.33	
ANC visit during pregnancy			
Four or more visits	1.00		0.131
One-three visits	1.38	0.96, 1.98	
No visit	1.83	0.96, 3.51	
Consumption of iron tablets during pregnancy			
Yes	1.00		0.199
No	1.39	0.84, 2.30	
Smoke			
No	1.00		0.247
Yes	1.47	0.75, 2.88	
Fuel			
Low polluting fuel	1.00		0.023*
Highly polluting fuel	1.56	1.07, 2.28	
Gestation and fetal growth factors			
Mother’s age at child’s birth (Years)			
≥ 30	1.00		0.970
25–29	0.91	0.55, 1.51	
20–24	0.92	0.56, 1.50	
15–19	0.99	0.60, 1.66	
Parity			
Four and above	1.00		0.748
Two-three	0.94	0.60, 1.48	
One	0.84	0.51, 1.38	
Gender of baby			
Male	1.00		0.379
Female	1.13	0.86, 1.48	

*p*-value was calculated from Wald test, *statistically significant at 5% level

The significant variables in univariate analysis were further analyzed by using a multiple survey logistic regression model. Adjusted odds ratio (OR) and its 95% CI are shown in Table 3. The inference statistical tests were nearly unchanged for the final model. Women with the lowest autonomy on their own health compared to those with involvement of husband or others (adjusted OR 1.87, 95% CI = 1.31, 2.67) and with husband and women together (adjusted OR 1.57, 95% CI = 1.05, 2.35) had a greater chance to give birth to LBW infants. For the other significant variable, mothers using highly polluting cooking fuels (adjusted OR 1.56, 95% CI = 1.03, 2.22) were more likely to give birth to LBW infants than mothers using non-polluting cooking fuels.

**Table 3:** Adjusted odds ratio and 95% CI of study variables

Variables	Adjusted OR	95% CI	*p*-value
Women’s decision for health service utilization			
Women	1.00		0.006*
Women and husband together	1.57	1.05, 2.35	
Husband or others	1.87	1.31, 2.67	
Fuel			
Low polluting fuel	1.00		0.045*
Highly polluting fuel	1.49	1.03, 2.22	

*p*-value was calculated from Wald test, *statistically significant at 5% level

## Discussion

The overall prevalence of LBW from this study is 15.4% which is different from the study including only infants with measured birth weight conducted by [15] in which the prevalence of LBW was found to be 11.5%. The difference is expected, because in this study there is an inclusion of additional 3,318 missing birth weight in the analysis. A study conducted by [14] found the prevalence of small size at birth as 16% which is close to the prevalence of this study. This may be because mother’s recall of infant’s size at birth and other variables are used for imputing missing values in this study. As shown in Table 1, the prevalences of LBW for the determinants like mother’s age at child’s birth, gender of child, residence, ethnicity and parity are almost equal in each subgroup. It can be concluded that each subgroup has equal chance of having LBW infants. In this study, the prevalences of having LBW infants are higher among mothers living in low standard such as being poor, using highly polluting cooking fuels, not attending ANC visit and not consuming iron tablets during pregnancy than those of their respective subgroups and this finding is consistent with the previous study conducted by [14].

The prevalences of LBW for BMI and ethnicity in each subgroup are surprisingly different from normal perception. For BMI, women with overweight have the lower prevalence and the lower odds of LBW compared to women with normal and underweight. The possible explanation for this is that overweight mothers are likely to give birth to bigger babies and underweight mothers are likely to give birth to smaller babies. This finding is consistent with the studies conducted by [32, 33]. Furthermore, the results of this study reveal that the prevalence of LBW among relatively advantaged mother is higher than relatively disadvantaged mother (janajati). Even though, there have been studies on ethnicity affecting on LBW, these studies were performed in the high income countries [34, 35]. From those studies, it seems that mothers from the advantaged group are less likely to give birth to LBW infants. However, in this study, the different effects on LBW from mothers with different ethnic backgrounds are insignificant because *p*-value is higher than 0.05 from unadjusted odds ratio. Therefore, it is inconclusive to state that the odds of having LBW infants from differently ethnic mothers can be distinguished.

The current study finds that a mother has higher odds to give birth to LBW babies, when her decision on utilization of health services is relied only on others instead of herself and this finding is supported by [36] in which women with the lowest decision making autonomy were more likely to have LBW. This is probably because women with the lowest decision making autonomy on their health care are less likely to receive regular health checkups together with ANC visit during pregnancy including safe deliveries and health information regarding pregnancy and childbirth. Apart from that, women with the lowest decision making autonomy on their own health may have poor nutrition uptake during pregnancy and that may consequently impair fetal growth [36]. The variables such as ANC visit during pregnancy and consumption of iron tablets during pregnancy are not significant with LBW in the current study. However, studies performed by [14, 15] found that mothers who did not attend ANC visit during pregnancy and mothers who did not consume iron tablets during pregnancy were more likely to give birth to LBW infants. This difference may be because [14, 15] assumed the missing values presented on ANC visit and iron tablets consumption during pregnancy as no ANC visit and no consumption of iron tablets during pregnancy respectively. The result from this study also finds that mothers who use highly polluting fuel are more likely to give birth to LBW infants and this finding is supported by a study conducted in India [37]. However, cooking fuel was found insignificant in the previous studies conducted in Nepal by [14, 15]. This is probably because [14, 15] supposed that mothers who did not belong to households (non dejure residents) used highly polluting cooking fuel.

The current study consists of missing data on the variables like birth weight, BMI, ANC visit, consumption of iron tablets during pregnancy, cooking fuel and women’s decision for utilization of health services. For birth weight, even though there has been a considerable rise in the percentage of measurement of infants birth weight at birth in the past 5 years from 17% in 2006 to 36% in 2011 [13, 38], but home delivery is still a preferred choice for most mothers in Nepal as stated in [39, 40]. Eventually, the problem of missing data on birth weight may continue for a long period. This suggests promoting and strengthening institutional delivery, provision of weighing scale and training to community health workers for measurement of birth weight of those infants who are born at home. However, missing data in other variables can be minimized with other measures. For instance, in DHS survey, the questions related to cooking fuel, collected in household level, should be assigned to individuals in the individual data file. Thus, a mother who is not member of household lack the data on cooking fuel and the problem of missing data on cooking fuel can be avoided, if questions related to cooking fuel are included in women’s questionnaire too.

Multiple imputation is employed in this study to handle missing data, because the analysis based on only complete cases of measured birth weight cannot be used since missing data are presented in more than one variable and the missing data are MAR. Moreover, using multiple imputation reduces bias downwards compared to analysis of complete cases, but it does not mean that using imputation methods for replacing missing values removes the bias completely.

The limitation of this study is that the efficiency of multiple imputation cannot be determined, because the data lack the completed record. Secondly, this efficiency might be lower because of high numbers of missing data. The study conducted by [7] mentioned that the results from statistical analysis are more prone to be biased, when the amount of missing is greater than 10%. However, as stated by [41], missing the data pattern and missing mechanism are more important than the percentage of missing data. Furthermore, the current study utilized the secondary data; thus, the exact reason for missing data is not clear for many variables.

## Conclusions

The findings of this study suggest that obtaining the prevalence of LBW from only the sample of measured birth weight results in underestimation of the prevalence. In addition, assuming missing values as non missing provides different results from the results with imputed data. Therefore, it is suggested for future researchers conducting studies on LBW with DHS data from low income countries that missing data on birth weight and its determinants should be imputed.

## Abbreviations

ANC: Antenatal care
BMI: Body mass index
CI: Confidence interval
DHS: Demographic and health survey
FCS: Fully conditional specification
LBW: Low birth weight
MAR: Missing at random
MCAR: Missing completely at random
MICE: Multiple imputation by chained equations
MID: Multiple imputation then deletion
MNAR: Missing not at random
NDHS: Nepal demographic and health survey
OR: Odds ratio
WHO: World health organization
